# Case report: Squamous cell carcinoma and spindle cell sarcoma (SCS) arising in a mature cystic teratoma of the ovary

**DOI:** 10.3389/fsurg.2023.1193994

**Published:** 2023-06-28

**Authors:** Xue-qian Qian, Li-li Chen, Chang-kun Zhu, Ya-xia Chen, Xiao-yun Wan

**Affiliations:** Department of Gynecologic Oncology, Women’s Hospital, School of Medicine, Zhejiang University, Hangzhou, China

**Keywords:** mature teratoma, spindle cell sarcoma, squamous cell carcinoma, CA199, malignant transformation

## Abstract

**Objective:**

Malignant transformation of mature ovarian teratoma is a rare phenomenon, mainly occurring in postmenopausal period. Squamous cell carcinoma accounts for 80% of all malignant transformations. Sarcoma transformation is much less common and tends to imply a poorer prognosis and aggressiveness.

**Case report:**

We report a case of undifferentiated sarcoma with squamous cell carcinoma in a mature cystic teratoma of the ovary in a 36-year-old woman. The tumor shows epithelial and stromal components. This is a unique report of a benign teratoma of the ovary with malignant transformation, showing epithelial and sarcomatous components. This young woman presented with abdominal distension and a rapidly enlarging ovario-derived pelvic mass with a slightly elevated CA199 tumor marker of 115.9 U/ml. The woman underwent transabdominal excision of the left ovarian cyst on October 20, 2020. During the operation, rapid freezing pathological examination did not indicate malignancy. The postoperative paraffin pathology revealed undifferentiated sarcoma with squamous cell carcinoma (from mature cystic teratoma malignancy), and she finally received comprehensive staging surgery. Postoperative paraffin pathology showed no residual cancer in uterus and other tissues, and all lymph nodes were negative. The patient was finally diagnosed with ovarian malignant tumor IC1 stage (high-grade spindle cell sarcoma complicated with squamous cell carcinoma). Chemotherapy was completed three times after surgery, and no signs of recurrence were found after follow-up.

**Conclusion:**

The preoperative diagnosis and intraoperative rapid freezing examination of malignant transformation of mature teratoma of ovary are challenging.

## 1. Introduction

Malignant transformation of mature teratoma is rare, mostly occurring in postmenopausal women and can be caused by any component of teratoma (1). Squamous cells are the most common malignancies, accounting for 80% of all malignant transformations, followed by adenocarcinoma and melanoma (2). Sarcoma transformation is much less common, with only a few rare reports in the literature, which often indicates a poor prognosis and a high degree of invasion. To improve the clinicians’ awareness of the disease, here we report an undifferentiated sarcoma with squamous cell carcinoma in a particularly young 36-year-old female mature cystic teratoma of the ovary.

## 2. Case presentation

A case of squamous cell carcinoma (SCC) and spindle cell sarcoma (SCS) arising from a mature cystic teratoma of the ovary in a 36-year-old woman was reported. The clinical features of the patient at baseline were summarized in Table 1. Follow-up time was 29 months.

**Table 1:** The clinical characteristics of the patient.

Clinical features of the patient	
Age (years)	36 (years)
Previous surgery	No
HPV	Negative
Gravida (times)	0
Para (times)	0
Metastasis	No
Symptoms	Abdominal distension pain
Tumor size (cm)	The diameter was 14 cm
Intraoperative rapid freezing pathology	This is a mature cystic teratoma with spindular cell proliferation in the local cyst wall with mild atypia.
First operation	Transabdominal excision of left ovarian cyst
Second operation	Total hysterectomy, adnexectomy and pelvic lymphotomy
SCC	2.9 ng/ml
CA199	115.9 U/ml

**Figure 1:**
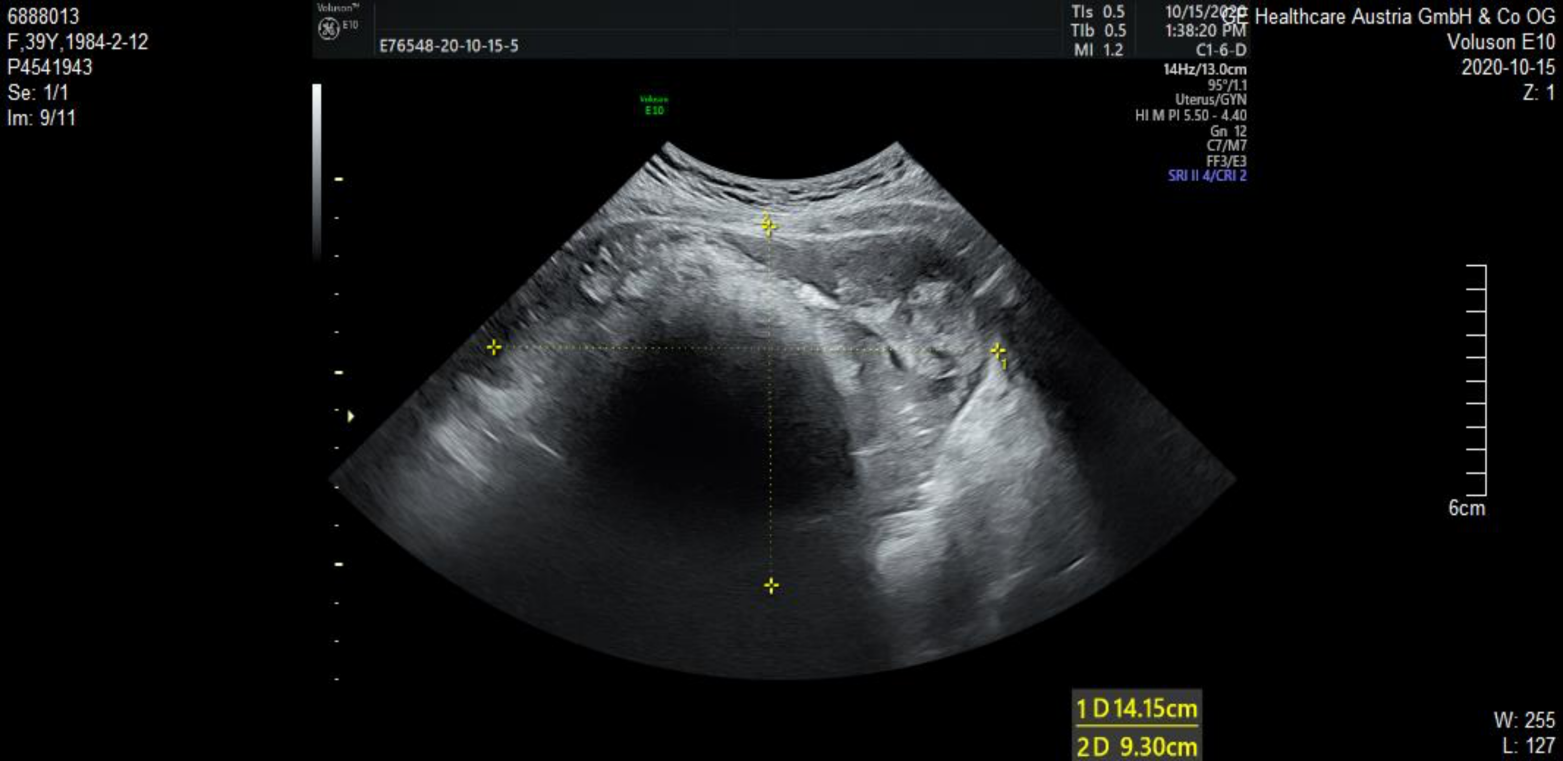
A pelvic mass examined by transvaginal ultrasound. Uneven echo mass of 14.2 cm × 9.3 cm × 12.8 cm was observed in the pelvic cavity, the boundary was clear, attenuation was observed in the rear, and no blood flow signal was observed.

In our case, the patient presented with short-term progressive abdominal distension and self-touching abdominal mass. Due to severe abdominal distension within a month, she came to our hospital for treatment. Physical examination revealed a large mass in the pelvic cavity, reaching umbilical level, with clear boundaries and no tenderness. The mass appeared fixed. No supraclavicular lymph nodes were involved and no abnormalities were observed on breast examination. Ultrasound examination indicated that there was an uneven echo mass of 14.2 cm × 9.3 cm × 12.8 cm in the pelvic cavity, the boundary was clear, attenuation was observed in the rear, and no blood flow signal was observed (Figure 1). Tumor markers only indicated an increase in CA199 (115.9 U/ml) and squamous cell carcinoma antigen (SCC) (2.9 ng/ml). Other tumour markers like AFP, beta HCG and LDH, which are associated with germ cell tumors (GCT), were also tested and showed to be normal.

The woman underwent transabdominal excision of the left ovarian cyst on October 20, 2020. A large solid cystic mass was found in the pelvic cavity, with a diameter of about 14 cm, smooth surface, solid tissue size of about 5.0 cm × 3.0 cm × 3.0 cm, and obvious vascular filling on the surface. No ascites or peritoneal deposits were found. An incision of about 1.0 cm was made on the surface of the left ovarian cyst, and a large number of hairs were found inside the cyst, with cephalic segment and solid crisp tissue. The cyst fluid was sucked, about 1,000 ml, pale yellow and thick. The cyst was extracted completely and sent for frozen pathological examination. The results showed mature cystic teratoma (left ovary), with spindle cell hyperplasia in local cyst wall accompanied by mild atypia.

**Figure 2:**
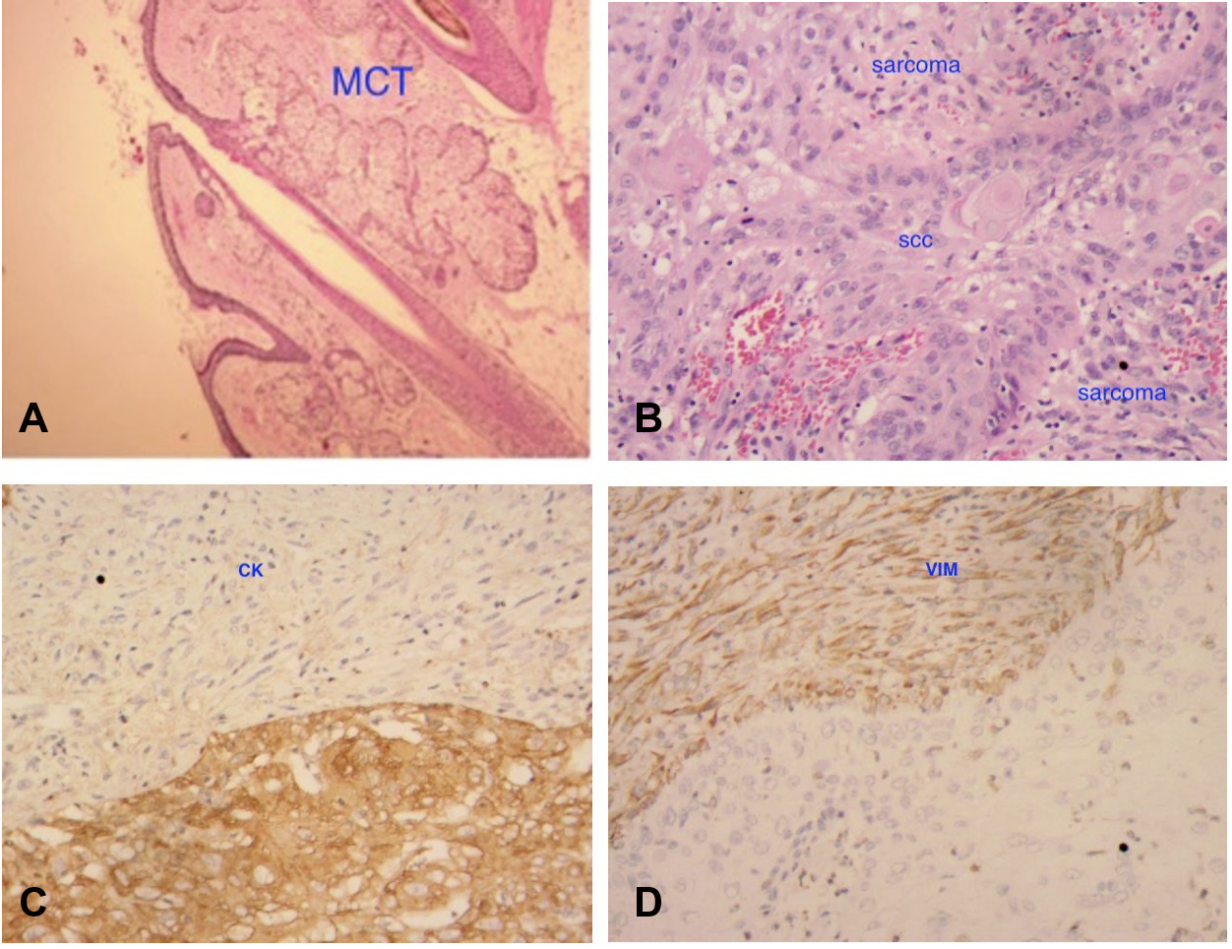
Histological finding at original magnification ×20. ① (**A**) Shows the sebaceous gland and appendages, suggesting mature cystic teratoma (MCT); ② (**B**) shows mixed two components (both squamous cell carcinoma and sarcoma); ③ (**C,D**) indicates that both CK and vimentin are positive, suggesting bidirectional tumor differentiation.

Postoperative routine paraffin pathology showed: (left ovary) high-grade spindle cell sarcoma, first considering pleomorphic undifferentiated sarcoma with squamous cell carcinoma (from mature cystic teratoma malignancy) (Figure 2). The immunohistochemical results are shown in Table 2. The positive rate of Ki-67 was 90%. CK and Vimentin reactions were positive, suggesting bidirectional differentiation toward epithelial and mesenchymal tumors, while other sarcoma differentiation lineage markers such as CK5/6(−), Desmin(−), SMA(−), HMB45, SOX-10(−) suggest undifferentiated sarcoma. Positive p53 is consistent with the diagnosis of squamous cell carcinoma.

**Table 2:** Immunohistochemical features.

Positive expression	Ki-67(+++)	Vimentin(+++)	P53(++)	CK(+)	CD31(+)
Negative expression	P40(−)	P63(−)	P16(−)	CK5/6(−)	SMA(−)
	Desmin(−)	SOX-10(−)	CD34(−)	SOX-10(−)	S-100(−)
	Myoglobin(−)	HMB45(−)	Calponin(−)		

Although the patient was young and unmarried, considering the high degree of malignancy and poor prognosis, the patient finally underwent radical surgery, i.e., hysterectomy, bilateral adenectomy, and pelvic lymphotomy.

The patient was finally diagnosed with ovarian malignant tumor IC1 stage. The combination of paclitaxel and carboplatin chemotherapy was administered every 3 weeks, and no signs of recurrence were found after follow-up.

## 3. Discussion

Malignant transformation of mature cystic teratoma of the ovary is rare, with less than 2% of teratoma undergoing this transformation (3). Squamous cells account for the majority of malignant changes, while mixed malignancies in mature cystic teratoma are extremely rare. Risk factors for malignancy include patient age over 45 years, tumor size, and tumor growth rate (4). Some studies have shown that mature cystic teratoma (MCTS) >100 mm in diameter are associated with an increased risk of malignancy (5). The mass in our patient was greater than 10 cm in diameter, which presented a high-risk factor for malignancy.

In patients with malignant transformation of teratoma, the typical clinical presentation is postmenopausal women aged 50–60 years (6), with abdominal pain, fullness, and constipation. This suggests that age may be a risk factor. The majority of patients were diagnosed with advanced stage (FIGO Stage II–III) and underwent total hysterectomy, bilateral oophorectomy, omentectomy, lymph node dissection, and cytoreductive surgery. The prognosis is poor, with most women dying within a year (7–9). The prospects for extraovarian diffusion are worse.

Through literature review, it was found that there were only two cases with mixed malignant components (shown in Table 3), one was a 58-year-old patient with squamous cell carcinoma and pleomorphic sarcoma (MFH), who progressed rapidly after diagnosis and survived for 5 months (10). Another case of multiple malignancies (squamous cell carcinoma and sarcoma) in a dermoid cyst of the ovary in a 75-year-old woman was still alive 21 months after surgery (11). Our patient, who was combined with both squamous cell carcinoma and sarcoma components, was the youngest case reported in the literature, and the tumor progressed significantly within a month, with progressive aggravation of abdominal distension, indicating rapid tumor progression.

**Table 3:** Summary of 2 reported cases of multiple malignancies in the English literature.

Case	Age	Symptom	Physical examination	Tumor marker	Treatment	Histopathology	Prognosis/Follow-up
Hanada et al. (8).	75	Pelvic mass	A large, suprapubic mass extending up to the level of the umbilicus was palpable in the right to mid-lower quadrant. The mass was movable, and lobular in shape with a cyst-like sensation.	Alpha-fetoprotein was 13.8 NG/ml (normal below 10 NG/ml)	A right salpingo-oophorectomy, with a complete resection of the tumor including the appendix and part of the omentum which had been hardly adherent to this tumor, was successfully performed.	Squamous cell carcinoma and sarcoma	Asymptomatic/21 (months)
Eleonora Savitchi et al. (9).	58	Left abdominal pain	A large complex cystic and solid left adnexal mass, measuring 18–20 cm. No ascites was noted.	Cancer antigen-125 was measured at 41 U/ml (reference 35 U/ml).	The patient underwent total abdominal hysterectomy with bilateral oophorectomy, omentectomy, appendectomy, pelvic lymph node dissection, and tumor debulking.	The tumor had 2 components: an epithelial component (squamous cell carcinoma) and a stromal component.	She was diagnosed with tumor recurrence and died soon thereafter, at 5 mo after the initial diagnosis.

For the preoperative diagnosis of teratoma malignancy, it is still difficult. For the imaging diagnosis of ovarian teratoma malignancy, there are usually papillae or solid components in the cyst or thickening of the cyst wall, which requires the attention of clinicians. Tumor markers CA125 and CA199 may be accompanied by a slight increase. It has been reported that squamous cell carcinoma antigen (SCC) can be used as a sensitive serum marker to distinguish squamous cell carcinoma from teratoma (12). In our patient, there was also a slight increase in CA199, which was consistent with literature reports.

The histogenesis of mixed tumor in female genital tract has always been controversial, and there are mainly two theories trying to explain it (10). The first theory is collision theory, and the second theory is binding theory, the former is double clonal tumor merger, and the latter assumes a common stem cell precursor. It is worth mentioning that both Ck and sarcomato-based markers such as vimentin were positive, so the possibility of carcinosarcoma should be on the alert. However, in carcinosarcoma, the carcinosarcoma and sarcoma components are more closely mixed, while in our patient the two components are separated, so our pathologist ruled out carcinosarcoma as a diagnosis.

Considering the poor prognosis of teratoma malignancies, early detection, early diagnosis and early treatment should be performed for teratoma patients. Our patient has been followed up for 29 months, no signs of recurrence, and the survival rate is higher than that reported in the literature. The possible reasons are related to the early stage and a comprehensive radical operation. However, it is worth drawing lessons that the rapid freezing of the patient during the operation did not indicate malignant lesions, and the possible factors include inadequate sampling, etc. Therefore, it is very important for the large tumor to obtain sufficient sampling.

## 4. Conclusion

The preoperative diagnosis and intraoperative rapid freezing of malignant changes caused by ovarian MT are challenging. Therefore, even young patients should be alert to the possibility of malignancy due to the large size and extensive solid composition of ovarian teratoma.

## Data Availability

The original contributions presented in the study are included in the article, further inquiries can be directed to the corresponding authors.
